# Spontaneous miscarriage driven by maternal genetic mutation at position of PAI-1-844G/A: shed light on a race-specific genetic polymorphism

**DOI:** 10.1186/s13104-023-06635-1

**Published:** 2023-12-06

**Authors:** Afrah Ameri, Khalil Khashei Varnamkhasti, Sara Parhoudeh, Samire Khashei Varnamkhasti, Leila Naeimi, Sirous Naeimi

**Affiliations:** 1 Department of Genetics, College of Science, Kazerun Branch, Islamic Azad University, Kazerun, Iran; 2 Department of Medical Laboratory Sciences, Faculty of Medicine, Kazerun Branch, Islamic Azad University, Kazerun, Iran

**Keywords:** Spontaneous miscarriage, *MTHFR* 1298 A > C, *MTHFR* 677 C > T, *Factor V Leiden* 1691 G > A, *PAI-1*-844G > A

## Abstract

**Objective:**

Association between a genetic polymorphism and disease, either positively or negatively, within a population may not necessarily predict association in other race-ethnic populations. The aim of this study was to genotype well recognized thrombophilia associated polymorphisms as common risk factors for miscarriage and investigate their benefit to use as risk factors in southwest region of Iran females (Khuzestan) in the Arabs ethnic minority group with spontaneous miscarriage. We developed a Reverse Dot Blot Assay for the genotyping of four polymorphisms.

**Results:**

There were significant differences in the genotype distribution and allelic frequencies of the *MTHFR* 1298 A > C, *MTHFR* 677 C > T, *Factor V Leiden* 1691 G > A, *PAI-1*-844G > A polymorphisms between the case and control groups. The *MTHFR* 1298 A > C, *MTHFR* 677 C > T and *Factor V Leiden* 1691 G > A polymorphisms were significantly associated with spontaneous miscarriage risk. Unlike some other race-ethnic populations, *PAI-1*-844G > A polymorphism was associated with risk of developing unplanned miscarriage in Iranian Arabs ethnic minority group females.

**Supplementary Information:**

The online version contains supplementary material available at 10.1186/s13104-023-06635-1.

## Introduction

Human reproduction still faced tremendous challenges, such as spontaneous pregnancy losses after successful conception. Around 5% of young women of childbearing age are experiencing two or more miscarriages in a row occurrence within 20 weeks of gestation with no known cause [1, 2]. Previous pregnancies and environmental factors have been found to account for pregnancy losses in habitual aborters [3, 4], however, acquired or inherited thrombophilia run a higher risk to add further to losses caused by recurrent miscarriage (RM) [5]. Traditionally, thrombophilic gene single nucleotide polymorphism (SNP) known to be a risk factor for RM, and persistently investigators are looking for the genetic polymorphisms that manifests itself as a risk of RM [6, 7]. It is worth noting that some thrombophilia associated gene polymorphisms have been investigated for susceptibility to RM, but their associations are not always consistent as its impact may vary from one ethnicity to the other. For instance, an association between the polymorphisms of *MTHFR* gene (*MTHFR* 1298 A > C and *MTHFR* 677 C > T) with recurrent pregnancy loss, was reported in some ethnics, comprising; Korean women [8], Chinese women [9], Syrian women [10], and East Asians [11]. The *MTHFR* A1298C (rs1801133), an A to C transition at nucleotide 1298, which results in the replacement of Glu-429 by alanine, and *MTHFR* C677T (rs1801133), a replacement from C to T of nucleotide 677, cause to the substitution of ala-222 by valine, are two most common polymorphisms in the gene encoding for methylenetetrahydrofolate reductase enzyme (*MTHFR* gene). These SNPs through the alteration structure of enzymes, reducing enzyme activity, Viz. irreversible conversion of 5,10-methylenetetrahydrofolate to 5-methyltetrahydrofolate, and leads to hyperhomocysteinemia during pregnancy which may directly damage the endothelium and influence placental function [12]. Whereas, contradictory findings have been also reported showing no statistical association between *MTHFR* polymorphisms and RM among Caucasian population [13]. Similarly, a significant association between RM and the carriers of FV Leiden (FVL) polymorphism (*FVL* G1691A) has been reported in Turkish and Bosnian women [7, 14]. However, some existing results could not find any association between the *FVL* G1691A genotype and RM in white Caucasian and Palestinian women [15, 16]. Factor V Leiden 1691 G > A, is a substitution of amino acid arginine by glutamine at position 506, which results in increased thrombin generation by induce conformational change the activated protein C cleavage site on factor V [17]. Moreover, the polymorphisms of the *PAI-1* gene have been also considered. *PAI-1* gene is encoded for plasminogen activator inhibitor-1, as a serine protease inhibitor protein belonging to Serpins broadly distributed family. Activity-and serum concentration-dependent genetic changes in *PAI-1*, may limit fibrinolysis, and by hypofibrinolysis, alternatively, elevated risk for thrombotic complications for women. Although, the findings from a previous study has demonstrated that *PAI-1*-844G > A haplotype was connected to hypofibrinolysis status, but, among the literature, no evidence have seen for an association between the *PAI-1*-844G > A gene polymorphism and RM risk [18–20]. Thus far, studies have been performed to evaluate the associations between thrombophilia associated polymorphisms and miscarriage within some ethnic groups. Given the possible diversity among the ethnic groups, we were prompted to perform this population-based case–control study in the Arabs ethnic minority group females.

## Methods

### Subjects

In this population-based case–control study, conducted on 300 women in the age group of 19–38 years, with and without previous miscarriage history (n = 150/each), all participants (Arabs minority race) were recruited at the Narges lab, Shahid Chamran University of Ahvaz (Jundi Shapur), Ahwaz, Khuzestan Province, Iran, between 2020 and 2022. The demographic and biochemical characteristics of women with RM and those with normal pregnancies are shown in Table 1. The definition of the American Society for Reproductive Medicine (ASRM) for RM (as two or more pregnancy losses) [21], was used as studies' inclusion cases' criterion and any ectopic pregnancies, mole and pregnancies with unknown localization, other obstetric complications which might be related to thrombotic changes (preeclampsia, fetal hypotrophy, preterm delivery, preterm placental ablation, intrauterine fetal death), thromboembolic disease, and chronic diseases were excluded. The control group included women with at least a positive history of uncomplicated pregnancy.

**Table1:** Demographic and biochemical characteristics of patients and the control group

Characteristics	Case	Control	*P*-value
Age (yr)	27.87 ± 0.314	27.87 ± 0.314	0.198
BMI (kg/m2)	26.18 ± 2.42	24.96 ± 3.71	0.204
Parity	1.14 + 1.22	1.32 + 1.41	0.742
Gravity	2.61 + 1.83	2.34 + 1.99	0.951
Consanguineous marriage	18.4%	16.2	0.799
Smoking	2.1%	1.8%	0.883
TSH (mlU/L)	1.49 ± 0.70	1.36 ± 0.51	0.443
Total cholesterol (mg/dl)	173.56 ± 24.22	168.29 ± 27.66	0.293
Triglyceride (mg/dl)	106.22 ± 52.69	99.14 ± 26.98	0.601
AST	18.48 ± 2.99	20.63 ± 7.25	0.479
ALT	23.69 ± 5.98	25.51 ± 10.97	0.296
ALP	68.71 ± 14.53	61.25 ± 10.68	0.311
Insulin (IU/L)	9.98 ± 4.99	7.712 ±	0.248
Hematocrit (%)	36.26 ± 2.17	35.11 ± 6.24	0.279
Hemoglobin (g/dl)	13.02 ± 1.12	12.73 ± 1.01	0.611
Platelet (K/ml)	269,000 ± 6.150	272,000 ± 8.200	0.701
Folate (ng/ml)	10 ± 11	13 ± 10.4	0.214
Uric acid (mg/dl)	3.14 ± 0.68	2.95 ± 0.81	0. 821

AST, Aspartate aminotransferase, ALT, Alanine transaminase, ALP, Alkaline phosphatase

### Hardy–Weinberg equilibrium

We first evaluated the Hardy–Weinberg equilibrium (HWE) by computing expected genotype values versus observed genotype values for all polymorphic loci to check whether the population was in Hardy–Weinberg equilibrium.

### Molecular analysis

Using Oligo 7 software (version 7.54, Molecular Biology Insights Inc., Cascade, CO, USA), we designed three sets of primers and probes (primer and probe sequences reported in Table 2) of the multiplex polymerase chain reaction assay to amplify the *MTHFR*, *PAI-1* and *Factor V Leiden* genes.

**Table 2:** Detailed information about the multiplex PCR primers and probes and annealing temperature

Name	Sequence	Annealing temperature (°C)
MTHFR-1298-F	TGGTCAGCTCCTCCCCTACA	60
MTHFR-1298-R	CACTTATCATTATCTTCGCACAGACG	
MTHFR-1298-Probe	Bio-ACCATTCCGGTTTGGTTCTCCC	
MTHFR-677-F	TTTGGGAAATCCCGAGTCAA	
MTHFR-677-R	TCTCCTTCCACGACGGAGGT	
MTHFR-677-Probe	Bio-GCGGAAGAATGTGTCAGCCTC	
PAI-1-F	TGCGAGCGATACGAAGTTCT	55
PAI-1-R	GAGAGTCACTTTTATTGGGAACCA	
PAI-1-Probe	Bio-CCGTGAAAGAATTATTTTTGTGTTTC	
Factor V Leiden-F	GAGTCCTGCTACAAGATTTCA	55
Factor V Leiden-R	CTGTCCAGGGATCTGCTCTT	
Factor V Leiden-Probe	Bio-CCTCTGGGCTAATAGGACTACTTCTAATCTG	

DNA was extracted from 5 ml peripheral blood, collected in EDTA tubes using a standard salt extraction protocol [22, 23].

Accurate determination of extracted DNA quality was considered as the pre-PCR assessment criterion by measuring concentration and purity using a UV spectrophotometer (Nanodrop spectrophotometer, Biochrom WPA Biowave II, UK). Measures for every sample were administered three times at room temperature following sufficient mixing of all samples.

Thermal cycler (Thermo-Fisher, UK) used to perform multiplex PCR amplification in a black 96-well plate. PCR amplification was carried out in a total volume of 25 μl. Table 3 shows the components used for each reaction. The cycling conditions were as follows: 94 °C/15 s, 60 °C/30 s (*MTHFR*), 55 °C/45 s (*PAI-1*) and 55 °C/40 s (*Factor V Leiden*) (Annealing temperature as in Table 2), and 72 °C/10 min (for 45 cycles). PCR products were run by standard electrophoresis on 1.5% agarose gel for 10 min and visualized on UV transilluminator.

**Table 3:** Master mix amplification of PCR reaction; component, concentration and volume

Component	Volume per reaction (μl)	Final concentration
Template DNA	1	
Each of the primers for *MTHFR*, *PAI-1* and *Factor V Leiden*	1	0.39 pmol
2 × Master Mix Red	12.5	1X
H2O	5.5	
Total volume	25	

**Fig. 1:**
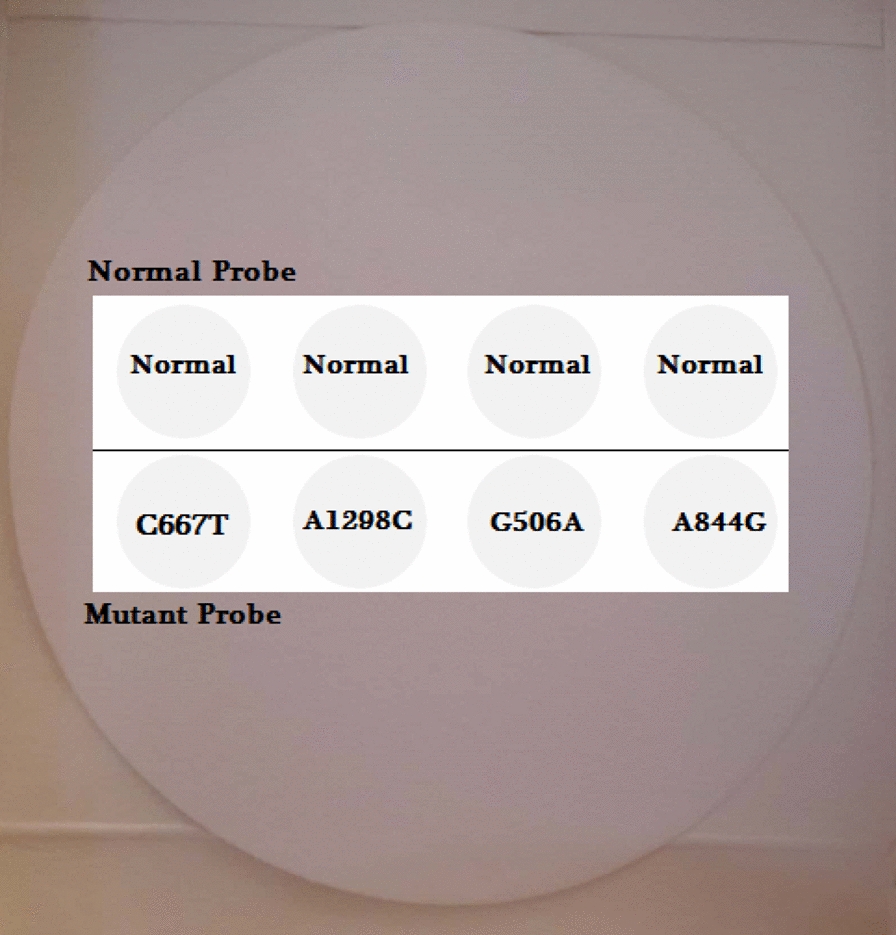
Location of normal and mutant probes on membrane

The reverse dot-blot method is a rapid diagnostic procedure that allows screening of sample for a variety of mutations/polymorphisms in a single hybridization reaction [24]. In this study, the reverse dot blot assay was evaluated based on membrane-fixed allele-specific oligonucleotides. The multiplexed PCR products were transferred in screw-top tubes with the strip and 10 ml of 2 × SSC and 0.1% SDS hybridization solution. After a denaturation (at 100 °C for 10 min) and incubated at 43.5 °C for 3 h steps, the strips washing in 0.5 × SSC and 0.1% SDS was carried out for 15 min at 43.5 °C. After hybridization and a washing step, strip incubation for 20 min at room temperature was performed by 10 ml of 2 × SSC, 0.1% SDS containing 5U of Streptavidin-POD conjugate (Roche, Mannheim, Germany). Excess conjugated was washed away in a 5-min washing step with the same buffer. The color was formed in the dark after addition of the 0.1 M sodium citrate (Takara, Dalian, China), pH 5.0, 0.1 mg/ml tetramethyl benzidine (Takara, Dalian, China) and 0.5 μl/ml 3% H2O2. Blue dot means test result is positive. Location of normal and mutant probes on membrane was depicted on Fig. 1.

### Sequencing

#### DNA template preparation

PCR fragments were purified from gel with the Favor PrepTM GEL/PCR purification kit (Favorgen Biotech Corp.; Ping-Tung, Taiwan) according to the manufacturer’s instructions. In this regard, DNA bands were excised from the agarose gel, and each band was transferred into a microcentrifuge tube. The gel slice was then properly dissolved by adding 500 μl of FADF Buffer and vortexing the tube every 2 ~ 3 min. Finally, the supernatant was carefully collected and was used as the template. The NanoDrop quantitative analysis was performed to check the purity of the DNA template.

#### PCR amplification of sequencing template

The PCR reaction mix was prepared according to the appropriate volumes per reaction in the Table 4, and the PCR program was run according to the thermal profile in the Table 5, using Genetic Analyzer 3130x (Applied Biosystems, USA). Sequences were analyzed with the CodonCode Aligner V.5.1.5 software (CodonCode Corporation, Centerville, MA, USA).

**Table 4:** Sanger Sequencing PCR reaction setup components

Component	Volume per reaction (μl)
Buffer big dye	3
Big-dye enzyme (BDT)	3
Primer	0.33
DW	7.67
DNA	6

**Table 5:** Sanger Sequencing PCR thermal profile

Cycles	Step	Temperature (°C)	Time
1 cycle	Initial Denaturation	94	5 min
	Denaturation	95	15 S
35 cycle	Annealing:		
	*MTHFR*	60	30 S
	*PAI-1*	55	45 S
	*Factor V Leiden*	55	40 S
	Extension	72	45 S
	Final Extension	72	10 min
	Hold	4	

#### Quality management in PCR runs and sequencing analysis

Apart from the quality and quantity of DNA and quality of reagents, primers, tubes and instrument calibration etc. the false positives were identified during the PCR runs and sequencing analysis by including non-target controls (reagent blank). If the reagent blank was being shown amplification of the target DNA the result of samples considered void.

### Statistical analysis

Statistical data analysis was performed using SPSS version 23 (SPSS Inc., Chicago, IL, USA). A Chi-square test (χ^2^) was used to analysis allele frequency and genotype frequency distribution between case and the control group, a *P*-value < 0.05 was considered statistically significant.

## Results

Checking for HWE showed that the deviation from HWE in the all polymorphic loci were not significant, therefore equilibrium was maintained for in question population at polymorphic MTHFR 1298 A > C (HWE *P*-value = 0.446), *MTHFR* 677 C > T (HWE *P*-value = 0.391), *Factor V Leiden* 1691 G > A (HWE *P*-value = 0.352), *PAI-1-844G* > *A* (HWE *P*-value = 0.402) sites.

**Fig. 2:**
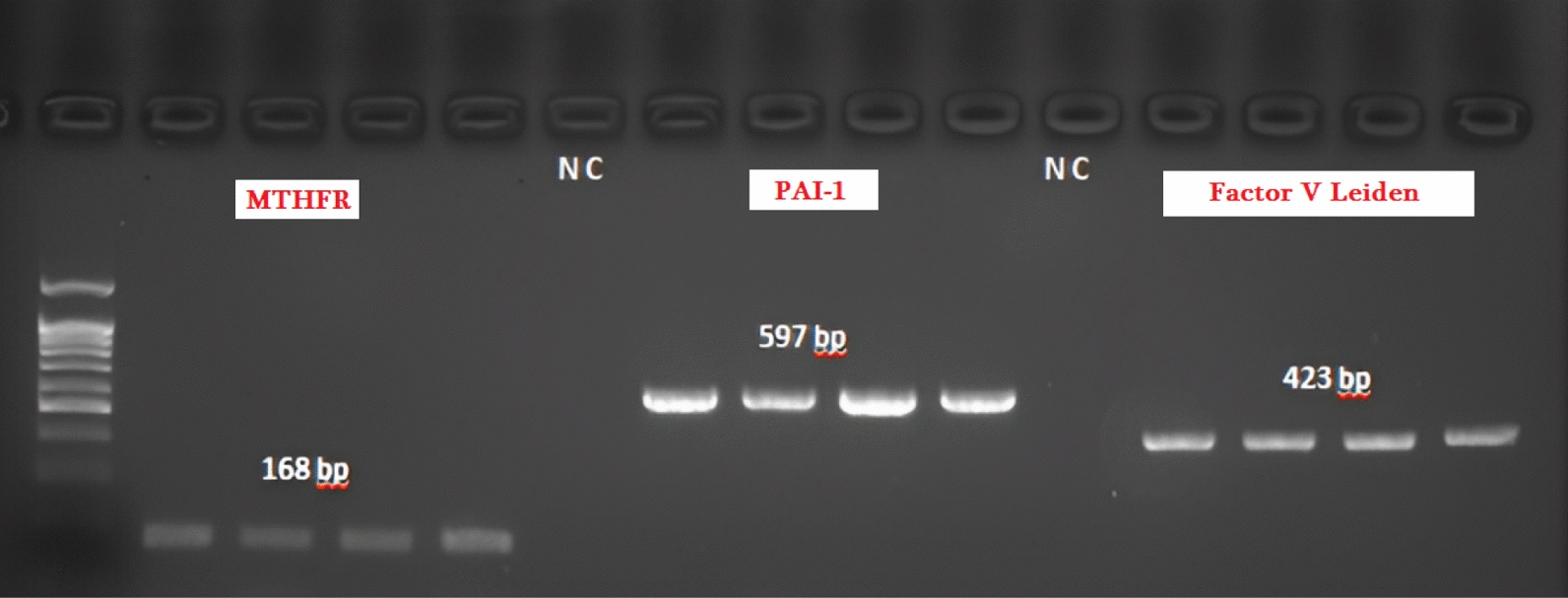
PCR amplification of the MTHFR, PAI-1 and Factor V Leiden genes

As shown in Fig. 2, *MTHFR* gene product length was 168 bp, *PAI-1* gene product length was 597 bp and *Factor V Leiden* gene had amplicon 423 bp in length.

**Fig. 3:**
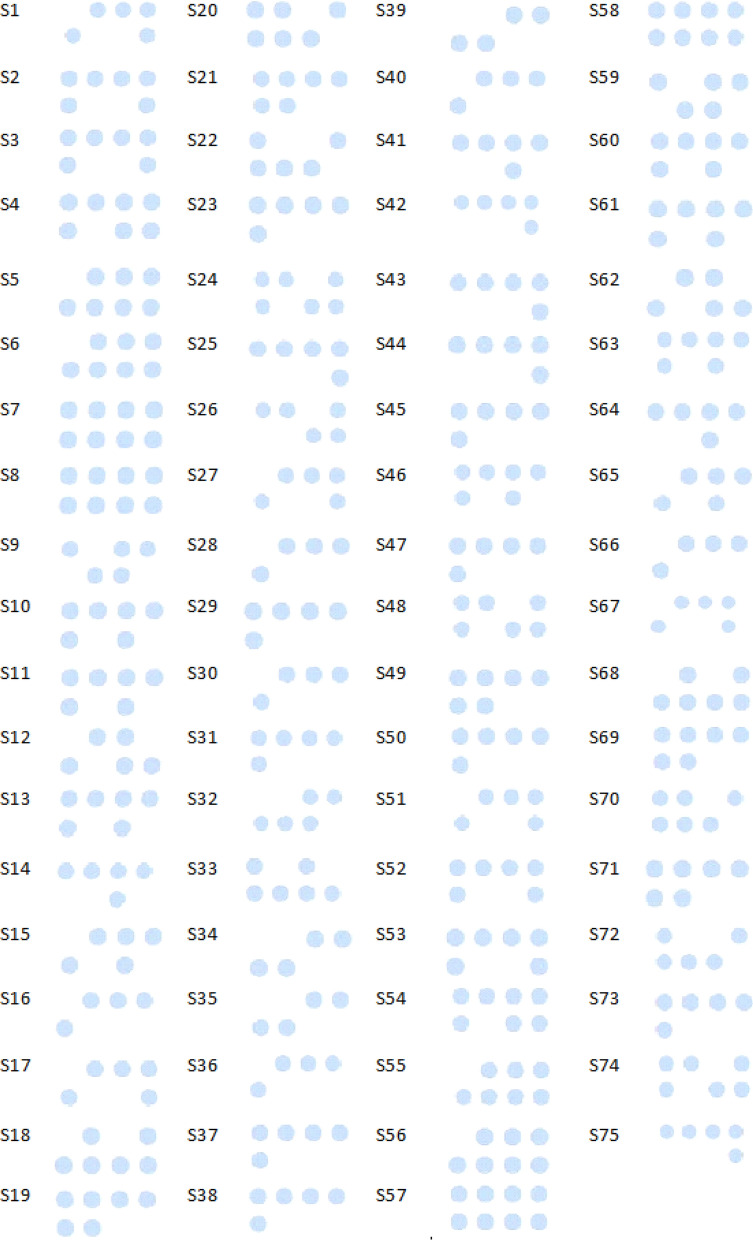
Results of the reverse dot blot assay for patients 1–75

**Fig. 4:**
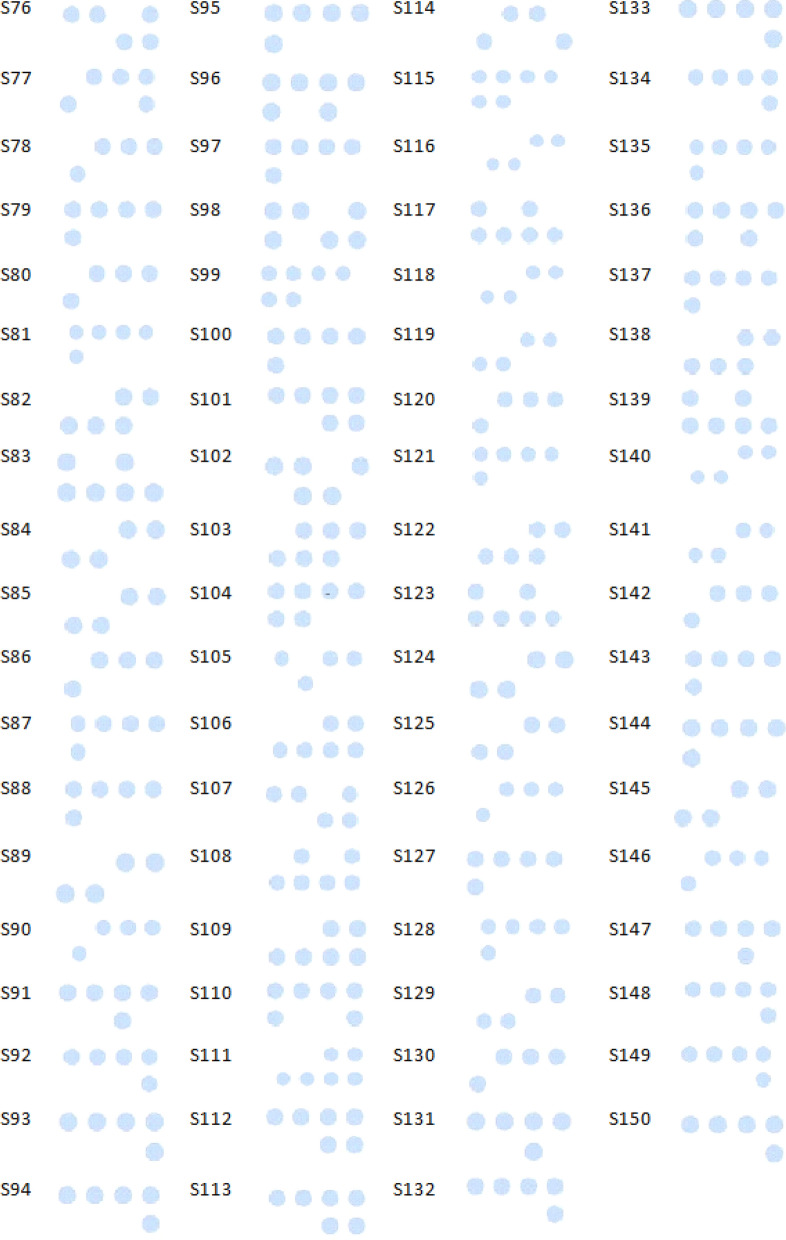
Results of the reverse dot blot assay for patients 76–150

Results from the reverse dot-blot test for the four thrombophilia associated polymorphisms are shown in Figs. 3 and 4. On each filter paper the normal probes were located above, and the mutant probes were below. For the Homozygous wild type pattern, DNA was hybridized to the normal probe but not to the mutant probe. In the Homozygous mutant pattern, DNA was only hybridized to the mutant probe. For Heterozygous DNA with a single copy of a mutation, the positive colored-spot for each of the probes (normal and mutant) was observed. Additional file 1 (as an unprocessed document) shows patients' genotype nature (Homozygous and heterozygous mutant or Homozygous wild type) with the *MTHFR* 1298 A > C, *MTHFR* 677 C > T, *Factor V Leiden* 1691 G > A, *PAI-1–844* G > A polymorphisms, also shown in Additional file 2: Table S1 accompanied with karyotype result and maternal age.

Table 6 shows the Genotype distribution and allelic frequencies of tested polymorphisms in the RM group and the control group. A significant difference in the distributions of the four polymorphisms was found among women with recurrent spontaneous abortions and controls (*P* < 0.05). The frequencies in the mutant allele of the *MTHFR* 1298 A > C, *MTHFR* 677 C > T, *Factor V Leiden* 1691 G > A, *PAI-1*-844G > A were statistically significantly higher in the RM than that in the control group (*P* < 0.05).

**Table 6:** Genotype distribution and allelic frequencies of the *MTHFR*, *PAI-1* and *Factor V Leiden* polymorphisms in case and control groups

Genotypes and alleles	Control group n (%)	Patient group n (%)	χ2	*P*-value
MTHFR 1298 A > C				
CC	21 (14)	69 (46)	0.985241	0.03
CA	47 (31.33)	44 (29.33)		
AA	82 (54.67)	37 (24.67)		
C	89 (29.66)	182 (60.66)	0.992144	0.01
A	211 (70.33)	118 (39.33)		
MTHFR 677 C > T				
CC	32 (21.34)	47 (31.33)	0.432125	0.02
CT	59 (39.33)	53 (35.33)		
TT	59 (39.33)	50 (33.34)		
C	123 (41)	147 (48.99)	0.875965	0.04
T	177 (58.99)	153 (51)		
Factor V Leiden 1691 G > A				
GG	28 (18.66)	72 (48)	0.954700	0.01
GA	30 (20)	57 (38)		
AA	92 (61.34)	21 (14)		
G	86 (28.66)	201 (67)	0.922548	0.02
A	214 (71.34)	99 (33)		
PAI-1-844G > A				
GG	42 (28)	27 (18)	0.915236	0.02
GA	61 (40.66)	49 (32.66)		
AA	47 (31.34)	74 (49.34)		
G	145 (48.33)	103 (34.33)	0.962145	0.02
A	155 (51.67)	197 (65.67)		

**Fig. 5:**
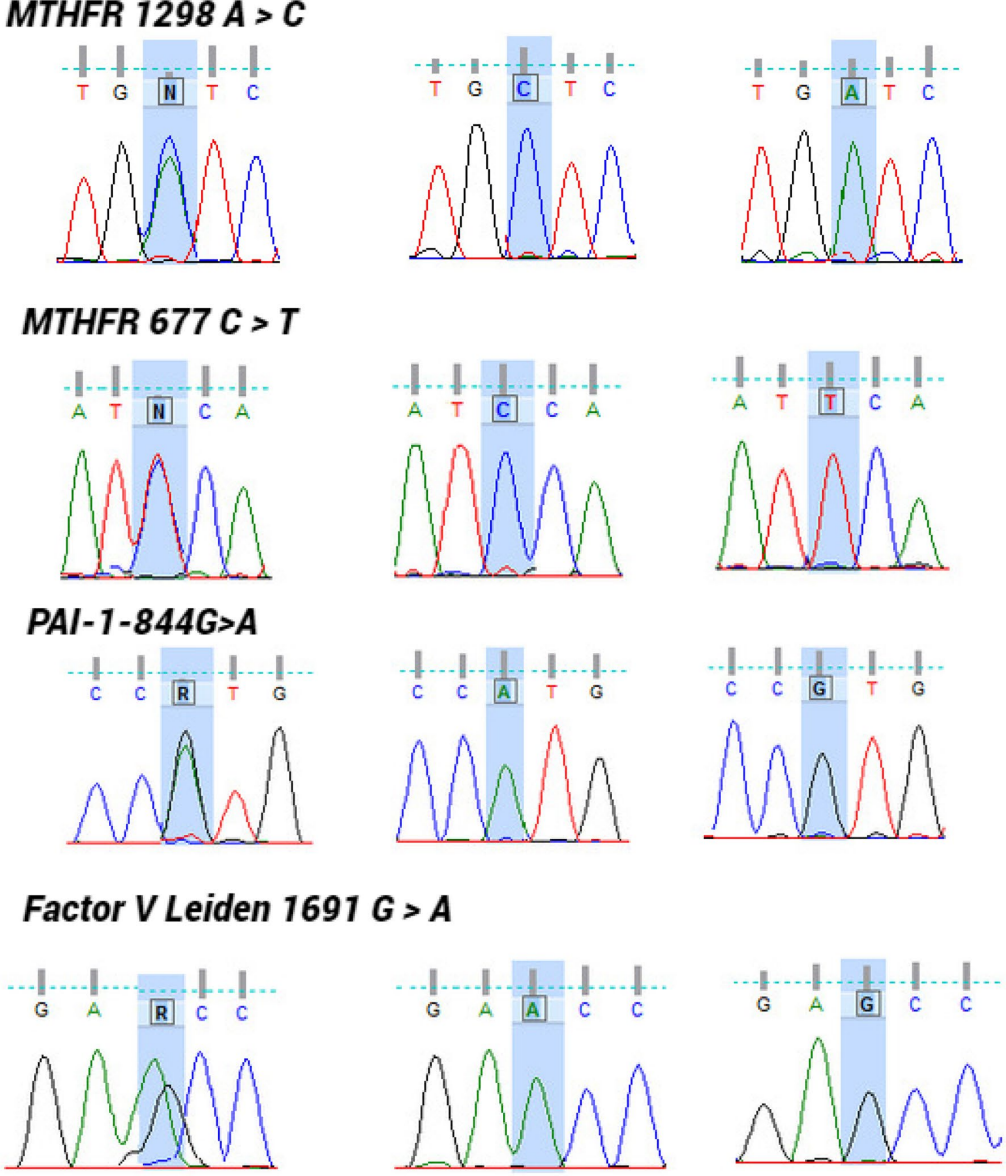
Result of DNA sequencing of PCR products of MTHFR 1298 A > C, MTHFR 677 C > T, PAI-1-844G > A and Factor V Leiden 1691 G > A polymorphisms, respectively

The accuracy and specificity of our established PCR was further validated by direct sequencing of PCR products. Figure 5 shows the PCR amplicon from genomic DNA, with a single peak in homozygous position (wild type pattern or mutant pattern). Homozygous wild type patterns are shown as A allele for *MTHFR 1298*, C allele for *MTHFR 677*, G allele for *PAI-1-844* and G allele for *Factor V Leiden 1691*. Moreover, the homozygous mutant patterns are shown as C allele for *MTHFR 1298*, T allele for *MTHFR 677*, A allele for *PAI-1-844* and A allele for *Factor V Leiden 1691*. Also Fig. 5 showing the results of the sequencing ran with a clear heterozygous pattern where both peaks (wild type and mutant) are present.

## Discussion

With the shifting health care trend toward precision medicine, the importance of race and ethnicity continue to be a highly investigated topic in medical research. Race and ethnicity diversity of participants is needed to help find a molecular biomarker that is generalizable to the disease. As discussed in the introduction, thus far, there are a plethora of publications on the role of thrombophilia gene polymorphism and its association with recurrent pregnancy loss, but a lack of uniformity in the studies available due to the ethnic group disparities is significant. As a result, it is unclear whether disease has a common molecular pathophysiology. Thus, further research into the relationship between race and polymorphism may potentially move a whole field of study forward. The present results expand the bedrock of progress toward generalizable biomarkers for RM genetic susceptibility. Such findings intensified the reliability of *MTHFR* 1298 A > C, *MTHFR* 677 C > T, and *Factor V Leiden* 1691 G > A, as generalizable markers for RM detection. In addition to that, we have found *PAI-1*-844G > A as a race-specific RM susceptibility locus for Arabs minority population. This can improve the current state of the scientific evidence and important scientific gaps in the literature on the RM of racial/ethnic minority groups. Although, some contemporary studies have confirmed the no association of − 844G/A with RM, such as those we mentioned in the introduction, the study by Zolfaghari et al., also suggests an influence of the − 844G/A genetic polymorphism on elevated risk for recurrent miscarriage [25]. As discussed, evidence-based consensus accepted by the entire scientific community is necessary for meaningful use of *MTHFR* 1298 A > C, *MTHFR* 677 C > T, *Factor V Leiden* 1691 G > A, *PAI-1*-844G > A, markers. Thus, the biomarker in a broader variety of cohorts that represent the full spectrum of disease should investigate. Some limitations of this study include: the sample size was relatively small, though it must be noted that similar studies had recruited a smaller sample of the participants [25]. The second limitation is ethnic homogeneity of the studied population, thus our results cannot be extrapolated to the general world population. Therefore, we recommend further investigation to limit the number of same loci whose frequency is greater across RM sufferer women from various ethnic subgroups.

## Conclusion

In conclusion, we reviewed the current state of the scientific evidence and identified that still there is a significant amount of heterogeneity between race and thrombophilia gene polymorphism prevalence among the population of recurrent miscarriage sufferers women from different countries. Therefore, more strive for generalizable *MTHFR* 1298 A > C, *MTHFR* 677 C > T, *Factor V Leiden* 1691 G > A, *PAI-1*-844G > A, as genetic markers for recurrent miscarriage-defining diagnostics across all racial/ethnic groups is needed.

### Supplementary Information

**Additional file 1.** Results from the reverse dot-blot test for the four thrombophilia associated polymorphisms.

**Additional file 2. Table S1**: Patients' genotype nature (Homozygous and heterozygous mutant or Homozygous wild type) with the MTHFR 1298 A > C, MTHFR 677 C > T, Factor V Leiden 1691 G > A, PAI-1-844G>A polymorphisms, karyotype result and maternal age.

## Data Availability

The data that supports the findings of this study are available within the article (and its Additional files 1 and 2).

## Abbreviations

RM: Recurrent miscarriage
SNP: Single nucleotide polymorphism
FVL: FV Leiden
ASRM: American Society of Reproductive Medicine
HWE: Hardy–Weinberg equilibrium
