# Supplementary figures and images for: Spontaneous miscarriage driven by maternal genetic mutation at position of PAI-1-844G/A: shed light on a race-specific genetic polymorphism
Source: BMC Res Notes. 2023 Dec 6;16:360. doi: 10.1186/s13104-023-06635-1 (PMC10702074; doi:10.1186/s13104-023-06635-1)

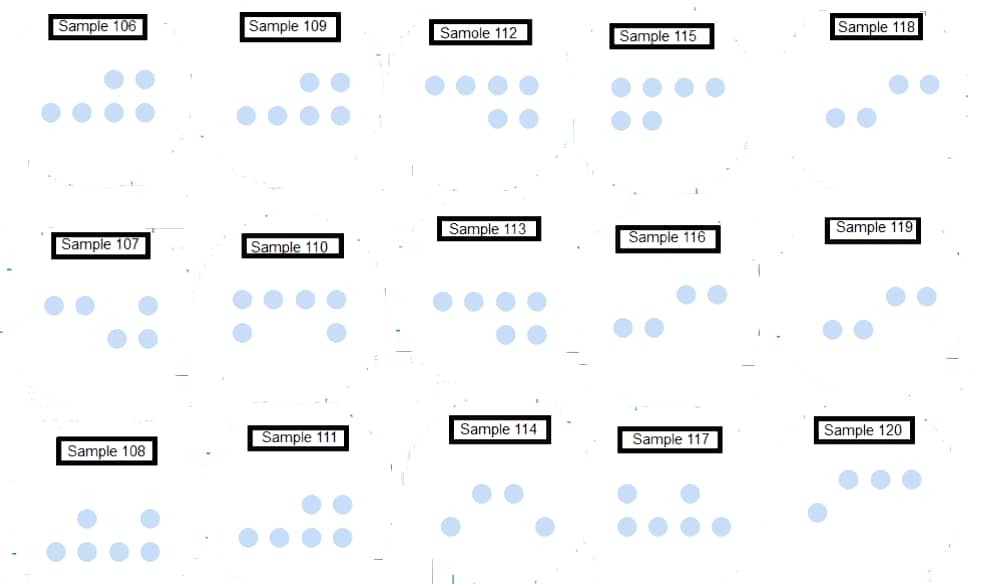

Supplement: Supplementary file 1 — Additional file 1. Results from the reverse dot-blot test for the four thrombophilia associated polymorphisms. [file 13104_2023_6635_MOESM1_ESM.zip › Sample 106-120.jpg]

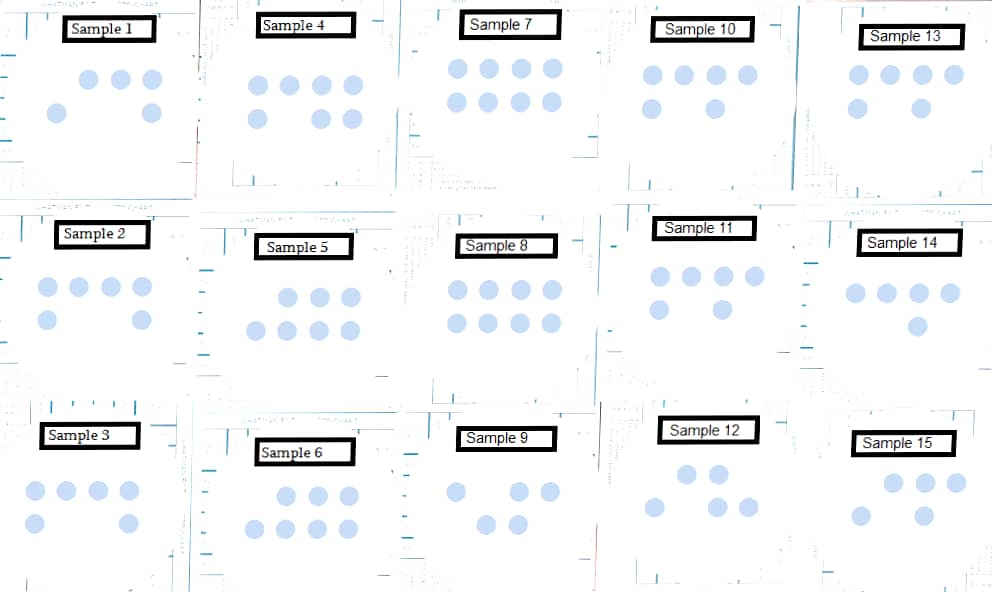

Supplement: Supplementary file 1 — Additional file 1. Results from the reverse dot-blot test for the four thrombophilia associated polymorphisms. [file 13104_2023_6635_MOESM1_ESM.zip › Sample 1-15.jpg]

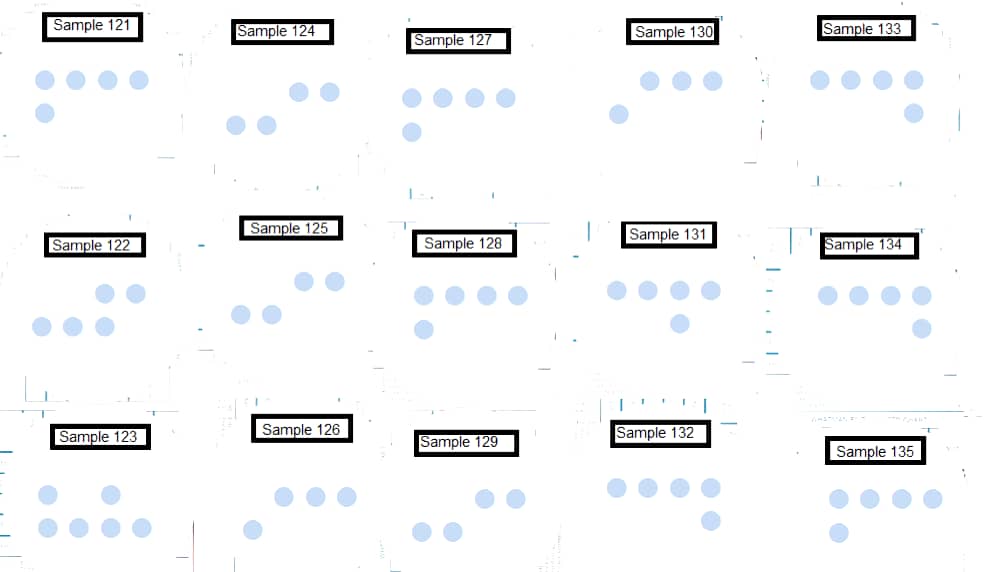

Supplement: Supplementary file 1 — Additional file 1. Results from the reverse dot-blot test for the four thrombophilia associated polymorphisms. [file 13104_2023_6635_MOESM1_ESM.zip › Sample 121-135.jpg]

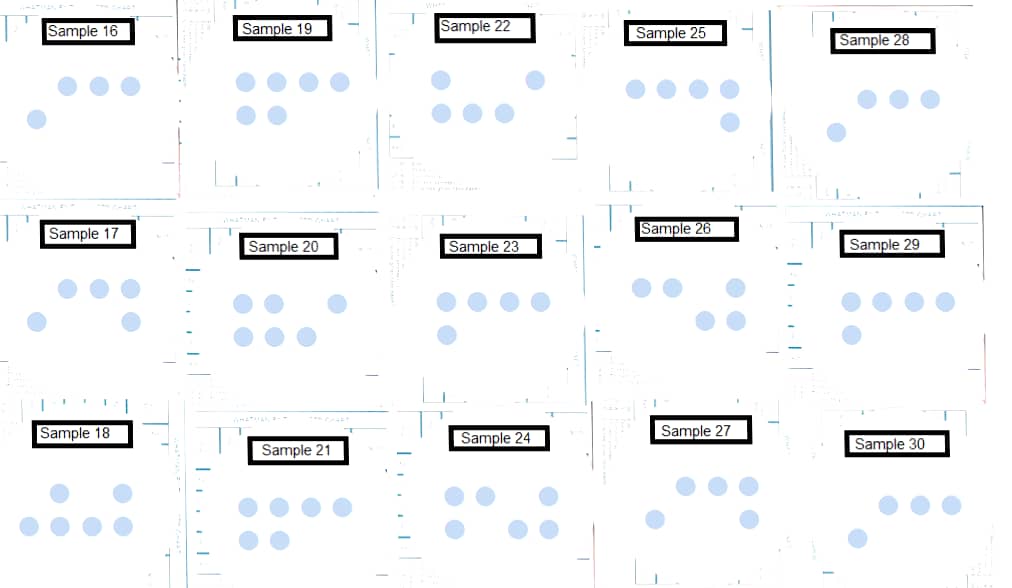

Supplement: Supplementary file 1 — Additional file 1. Results from the reverse dot-blot test for the four thrombophilia associated polymorphisms. [file 13104_2023_6635_MOESM1_ESM.zip › Sample 16-30.jpg]

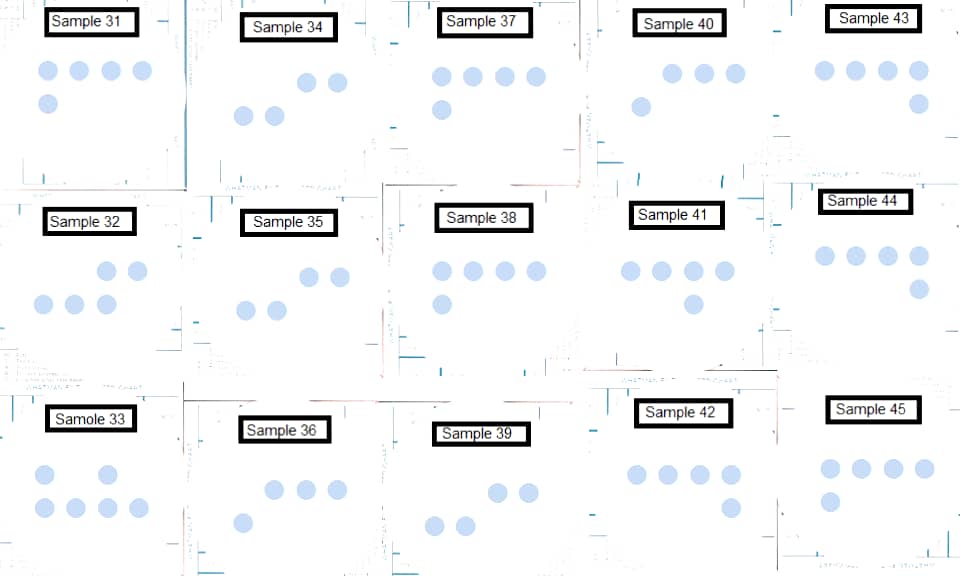

Supplement: Supplementary file 1 — Additional file 1. Results from the reverse dot-blot test for the four thrombophilia associated polymorphisms. [file 13104_2023_6635_MOESM1_ESM.zip › Sample 31-45.jpg]

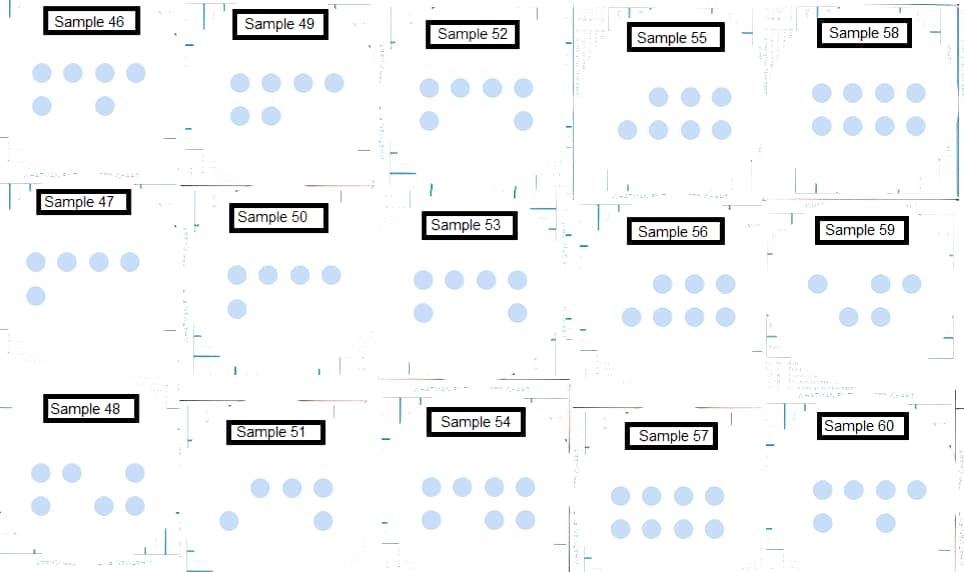

Supplement: Supplementary file 1 — Additional file 1. Results from the reverse dot-blot test for the four thrombophilia associated polymorphisms. [file 13104_2023_6635_MOESM1_ESM.zip › Sample 46-60.jpg]

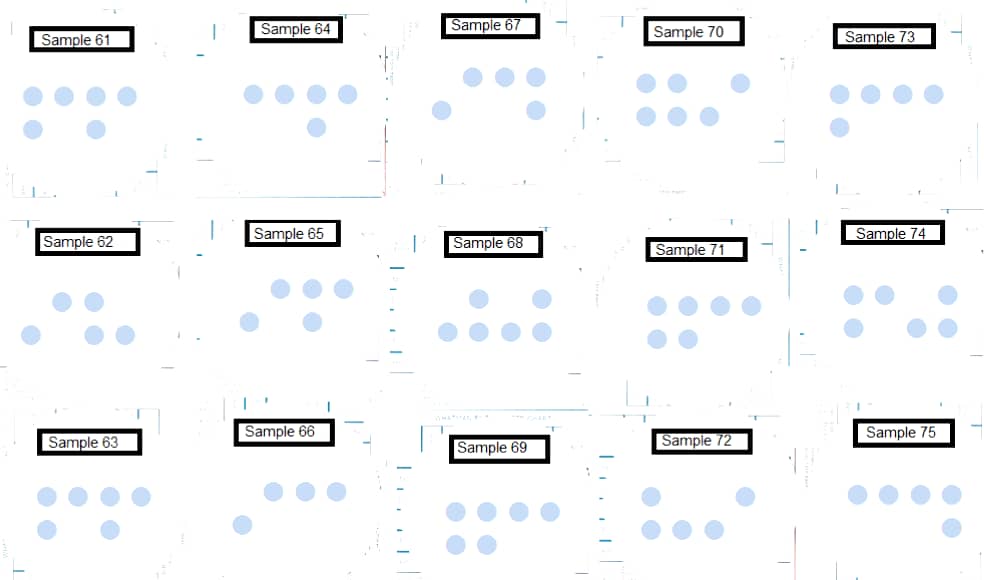

Supplement: Supplementary file 1 — Additional file 1. Results from the reverse dot-blot test for the four thrombophilia associated polymorphisms. [file 13104_2023_6635_MOESM1_ESM.zip › Sample 61-75.jpg]

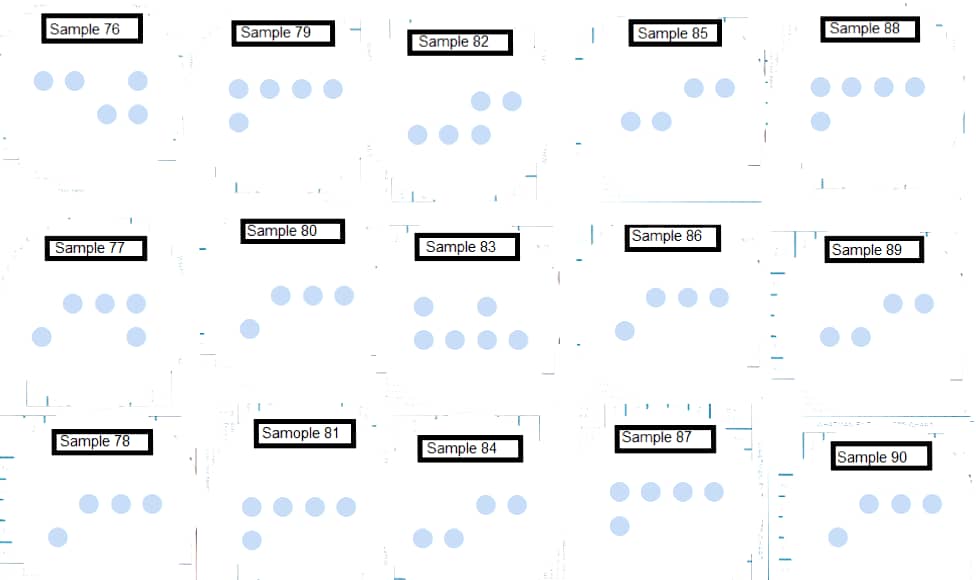

Supplement: Supplementary file 1 — Additional file 1. Results from the reverse dot-blot test for the four thrombophilia associated polymorphisms. [file 13104_2023_6635_MOESM1_ESM.zip › Sample 76-90.jpg]

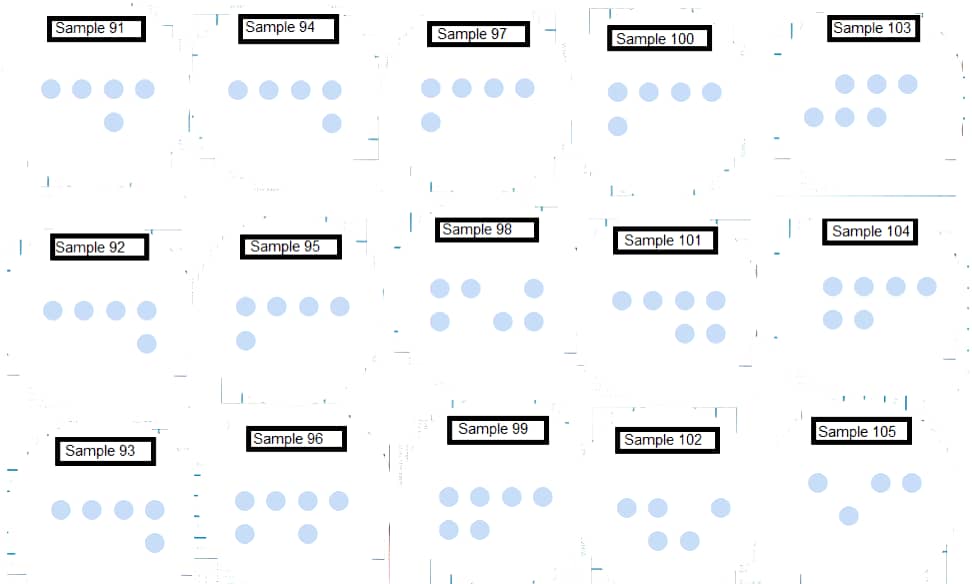

Supplement: Supplementary file 1 — Additional file 1. Results from the reverse dot-blot test for the four thrombophilia associated polymorphisms. [file 13104_2023_6635_MOESM1_ESM.zip › Sample 91-105.jpg]

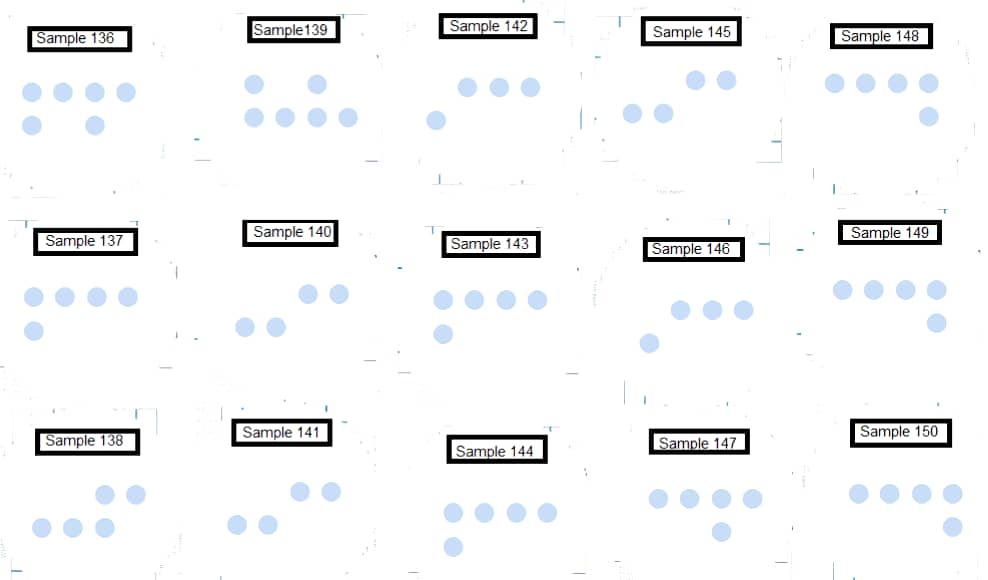

Supplement: Supplementary file 1 — Additional file 1. Results from the reverse dot-blot test for the four thrombophilia associated polymorphisms. [file 13104_2023_6635_MOESM1_ESM.zip › Sample136-150.jpg]
