# Supplementary material for: Spontaneous miscarriage driven by maternal genetic mutation at position of PAI-1-844G/A: shed light on a race-specific genetic polymorphism
Source: BMC Res Notes. 2023 Dec 6;16:360. doi: 10.1186/s13104-023-06635-1 (PMC10702074; doi:10.1186/s13104-023-06635-1)
Supplement: Supplementary file 2 — Additional file 2. Table S1: Patients' genotype nature (Homozygous and heterozygous mutant or Homozygous wild type) with the MTHFR 1298 A > C, MTHFR 677 C > T, Factor V Leiden 1691 G > A, PAI-1-844G>A polymorphisms, karyotype result and maternal age. [file 13104_2023_6635_MOESM2_ESM.docx]

Supplementary Table1 Patients' genotype nature (Homozygous and heterozygous mutant or Homozygous wild type) with the MTHFR 1298 A > C, MTHFR 677 C > T, Factor V Leiden 1691 G > A, PAI-1-844G>A polymorphisms, karyotype result and maternal age

|  | **MTHFR (C667T)** | **MTHFR**  **(A1298C)** | **FV**  **(G506A)** | **PA-1**  **(A844G)** | **MISCARRIAGE** | **KARYOTYPE** | **AGE** |
| --- | --- | --- | --- | --- | --- | --- | --- |
| 1 | M | W | W | H | 2 | 46، XX | 34 |
| 2 | H | W | W | H | 2 | 46، XX | 30 |
| 3 | H | W | W | H | 3 | 46، XX | 28 |
| 4 | H | W | H | H | 4 | 46، XX | 26 |
| 5 | M | H | H | H | 2 | 46، XX | 25 |
| 6 | M | H | H | H | 2 | 46، XX | 24 |
| 7 | H | H | H | H | 2 | 46، XX | 31 |
| 8 | H | H | H | H | 3 | 46، XX | 34 |
| 9 | W | M | H | W | 3 | 46، XX | 33 |
| 10 | H | W | H | W | 6 | 46، XX | 29 |
| 11 | H | W | H | W | 3 | 46، XX | 25 |
| 12 | M | W | H | M | 7 | 46، XX | 31 |
| 13 | H | W | H | W | 3 | 46، XX | 30 |
| 14 | W | W | H | W | 2 | 46، XX | 30 |
| 15 | M | W | H | W | 2 | 46، XX | 22 |
| 16 | M | W | W | W | 2 | 46، XX | 26 |
| 17 | M | W | W | H | 2 | 46، XX | 18 |
| 18 | M | H | M | H | 2 | 46، XX | 22 |
| 19 | H | H | W | W | 2 | 46، XX | 24 |
| 20 | H | H | M | W | 2 | 46، XX | 23 |
| 21 | H | H | W | W | 2 | 46، XX | 25 |
| 22 | H | M | M | W | 2 | 46، XX | 30 |
| 23 | H | W | W | W | 2 | 46، XX | 30 |
| 24 | H | W | M | H | 2 | 46، XX | 32 |
| 25 | W | W | W | H | 2 | 46، XX | 36 |
| 26 | W | W | M | H | 2 | 46، XX | 35 |
| 27 | M | W | W | H | 2 | 46، XX | 24 |
| 28 | M | W | W | W | 2 | 46، XX | 23 |
| 29 | H | W | W | W | 3 | 46، XX | 20 |
| 30 | M | W | W | W | 2 | 46، XX | 32 |
| 31 | H | W | W | W | 2 | 46، XX | 30 |
| 32 | M | M | H | W | 2 | 46، XX | 25 |
| 33 | H | M | H | M | 2 | 46، XX | 27 |
| 34 | M | M | W | W | 2 | 46، XX | 29 |
| 35 | M | M | W | W | 2 | 46، XX | 30 |
| 36 | M | W | W | W | 3 | 46، XX | 24 |
| 37 | H | W | W | W | 2 | 46، XX | 22 |
| 38 | H | W | W | W | 3 | 46، XX | 25 |
| 39 | M | M | W | W | 2 | 46، XX | 28 |
| 40 | M | W | W | W | 2 | 46، XX | 28 |
| 41 | W | W | H | W | 2 | 46، XX | 31 |
| 42 | W | W | W | H | 2 | 46، XX | 27 |
| 43 | W | W | W | H | 3 | 46، XX | 32 |
| 44 | W | W | W | H | 2 | 46، XX | 33 |
| 45 | H | W | W | W | 2 | 46، XX | 24 |
| 46 | H | W | H | W | 2 | 46، XX | 27 |
| 47 | H | W | W | W | 2 | 46، XX | 27 |
| 48 | H | W | M | H | 3 | 46، XX | 29 |
| 49 | H | H | W | W | 2 | 46، XX | 30 |
| 50 | H | W | W | W | 2 | 46، XX | 34 |
| 51 | M | W | W | H | 3 | 46، XX | 31 |
| 52 | H | W | W | H | 2 | 46، XX | 30 |
| 53 | H | W | W | H | 3 | 46، XX | 33 |
| 54 | H | W | H | H | 2 | 46، XX | 27 |
| 55 | M | H | H | H | 2 | 46، XX | 29 |
| 56 | M | H | H | H | 2 | 46، XX | 25 |
| 57 | H | H | H | H | 2 | 46، XX | 22 |
| 58 | H | H | H | H | 2 | 46، XX | 29 |
| 59 | W | M | H | W | 3 | 46، XX | 31 |
| 60 | H | W | H | W | 2 | 46، XX | 34 |
| 61 | H | W | H | W | 2 | 46، XX | 26 |
| 62 | M | W | H | M | 2 | 46، XX | 29 |
| 63 | H | W | H | W | 2 | 46، XX | 24 |
| 64 | W | W | H | W | 2 | 46، XX | 25 |
| 65 | M | W | H | W | 2 | 46، XX | 26 |
| 66 | M | W | W | W | 2 | 46، XX | 25 |
| 67 | M | W | W | H | 2 | 46، XX | 28 |
| 68 | M | H | M | H | 3 | 46، XX | 31 |
| 69 | H | H | W | W | 3 | 46، XX | 32 |
| 70 | H | H | M | W | 2 | 46، XX | 30 |
| 71 | H | H | W | W | 2 | 46، XX | 30 |
| 72 | H | M | M | W | 2 | 46، XX | 32 |
| 73 | H | W | W | W | 4 | 46، XX | 27 |
| 74 | H | W | M | H | 2 | 46، XX | 27 |
| 75 | W | W | W | H | 2 | 46، XX | 23 |
| 76 | W | W | M | H | 2 | 46، XX | 29 |
| 77 | M | W | W | H | 2 | 46، XX | 29 |
| 78 | M | W | W | W | 2 | 46، XX | 21 |
| 79 | H | W | W | W | 2 | 446، XX | 25 |
| 80 | M | W | W | W | 2 | 46، XX | 26 |
| 81 | H | W | W | W | 2 | 46، XX | 29 |
| 82 | M | M | H | W | 3 | 46، XX | 30 |
| 83 | H | M | H | M | 2 | 46، XX | 31 |
| 84 | M | M | W | W | 3 | 46، XX | 34 |
| 85 | M | M | W | W | 3 | 46، XX | 33 |
| 86 | M | W | W | W | 2 | 46، XX | 31 |
| 87 | H | W | W | W | 2 | 46، XX | 30 |
| 88 | H | W | W | W | 2 | 46، XX | 25 |
| 89 | M | M | W | W | 2 | 46، XX | 24 |
| 90 | M | W | W | W | 2 | 46، XX | 24 |
| 91 | W | W | H | W | 2 | 46، XX | 27 |
| 92 | W | W | W | H | 2 | 46، XX | 29 |
| 93 | W | W | W | H | 2 | 46، XX | 31 |
| 94 | W | W | W | H | 2 | 46، XX | 32 |
| 95 | H | W | W | W | 2 | 46، XX | 33 |
| 96 | H | W | H | W | 2 | 46، XX | 35 |
| 97 | H | W | W | W | 2 | 46، XX | 33 |
| 98 | H | W | M | H | 3 | 46، XX | 32 |
| 99 | H | H | W | W | 2 | 46، XX | 29 |
| 100 | H | W | W | W | 3 | 46، XX | 27 |
| 101 | W | W | H | H | 2 | 46، XX | 28 |
| 102 | W | H | M | W | 2 | 46، XX | 25 |
| 103 | M | H | H | W | 2 | 46، XX | 26 |
| 104 | H | H | W | W | 2 | 46، XX | 23 |
| 105 | W | M | W | W | 3 | 46، XX | 31 |
| 106 | M | M | H | H | 2 | 46، XX | 26 |
| 107 | W | W | M | H | 2 | 46، XX | 26 |
| 108 | W | H | W | H | 2 | 46، XX | 25 |
| 109 | M | M | H | H | 3 | 46، XX | 23 |
| 110 | H | W | W | H | 2 | 46، XX | 22 |
| 111 | M | M | H | H | 2 | 46، XX | 28 |
| 112 | W | W | H | H | 2 | 46، XX | 31 |
| 113 | W | W | H | H | 4 | 46، XX | 32 |
| 114 | M | W | W | M | 6 | 46، XX | 28 |
| 115 | H | H | W | W | 2 | 46، XX | 25 |
| 116 | M | M | W | W | 3 | 46، XX | 29 |
| 117 | H | M | H | M | 3 | 46، XX | 28 |
| 118 | M | M | W | W | 2 | 46، XX | 31 |
| 119 | M | M | W | W | 2 | 46، XX | 33 |
| 120 | M | W | W | W | 4 | 46، XX | 31 |
| 121 | H | W | W | W | 2 | 46، XX | 27 |
| 122 | M | M | H | W | 6 | 46، XX | 21 |
| 123 | H | M | H | M | 4 | 46، XX | 19 |
| 124 | M | M | W | W | 2 | 46، XX | 35 |
| 125 | M | M | W | W | 2 | 46، XX | 22 |
| 126 | M | W | W | W | 2 | 46، XX | 22 |
| 127 | H | W | W | W | 4 | 46، XX | 22 |
| 128 | H | W | W | W | 6 | 46، XX | 25 |
| 129 | M | M | W | W | 2 | 46، XX | 31 |
| 130 | M | W | W | W | 2 | 46، XX | 32 |
| 131 | W | W | H | W | 2 | 46، XX | 29 |
| 132 | W | W | W | H | 2 | 46، XX | 33 |
| 133 | W | W | W | H | 2 | 46، XX | 29 |
| 134 | W | W | W | H | 2 | 46، XX | 22 |
| 135 | H | W | W | W | 2 | 46، XX | 21 |
| 136 | H | W | H | W | 3 | 46، XX | 28 |
| 137 | H | W | W | W | 6 | 46، XX | 28 |
| 138 | M | M | H | W | 3 | 46، XX | 25 |
| 139 | H | M | H | M | 2 | 46، XX | 26 |
| 140 | M | M | W | W | 2 | 46، XX | 31 |
| 141 | M | M | W | W | 2 | 46، XX | 33 |
| 142 | M | W | W | W | 2 | 46، XX | 29 |
| 143 | H | W | W | W | 2 | 46، XX | 20 |
| 144 | H | W | W | W | 3 | 46، XX | 28 |
| 145 | M | M | W | W | 2 | 46، XX | 23 |
| 146 | M | W | W | W | 2 | 46، XX | 24 |
| 147 | W | W | H | W | 5 | 46، XX | 30 |
| 148 | W | W | W | H | 2 | 46، XX | 27 |
| 149 | W | W | W | H | 2 | 46، XX | 29 |
| 150 | W | W | W | H | 2 | 46، XX | 30 |

W: Homozygous wild type, M: Homozygous mutant, H: Heterozygous
